# Single VHH-directed BCMA CAR-T cells cause remission of relapsed/refractory multiple myeloma

**DOI:** 10.1038/s41375-021-01269-3

**Published:** 2021-05-24

**Authors:** Lu Han, Ji-Shuai Zhang, Jian Zhou, Ke-Shu Zhou, Ben-Ling Xu, Lin-Lin Li, Bai-Jun Fang, Qing-Song Yin, Xing-Hu Zhu, Hu Zhou, Xu-Dong Wei, Hong-Chang Su, Bing-Xiang Zhang, Ya-Nan Wang, Bin Xiang, Quan-Li Gao, Yong-Ping Song

**Affiliations:** 1 Department of Immunology, Affiliated Cancer Hospital of Zhengzhou University and Henan Cancer Hospital, Zhengzhou, 450008 Henan China; 2 The Shenzhen Pregene Biopharma Company, Ltd., Shenzhen, 518118 Guangdong China; 3 Department of Hematology, Affiliated Cancer Hospital of Zhengzhou University and Henan Cancer Hospital, Zhengzhou, 450008 Henan China

**Keywords:** Cancer immunotherapy, Immunotherapy

## To the Editor:

Chimeric antigen receptor T (CAR-T) cells have shown remarkable effects in treating hematological malignancies [1, 2]. Typically, the antigen recognition domain of CAR-T cells is a single-chain variable fragment (scFv) linked to a costimulatory domain and a cytoplasmic activation domain, such as CD28, 4-1BB, and CD3ζ [3, 4]. The scFvs are composed of a heavy-chain variable fragment connected to a light-chain variable fragment by a flexible linker optimized to preserve the pairing of heavy- and light-chain variable regions. These are usually derived from a full-length mouse immunoglobulin and can lead to human anti-mouse immune response. This immunogenicity can lead to adverse events and loss of efficacy during CAR therapy [5, 6]. In addition, scFvs do not always fold efficiently and can be prone to aggregation [7, 8].

As an alternative to scFvs, nanobody may serve as suitable antigen recognition domains in CAR-T cells. Nanobody (also called variable domain of heavy chain of heavy-chain antibody, VHH), is the variable fragment of heavy-chain antibodies of Camelidae. The heavy-chain antibodies are composed of only two heavy chains, with no light chain, but have the function of conventional antibodies. The VHH is the small, stable, single domain structure with high affinity and specificity comparable to those of scFvs [9, 10] and is easy to be humanized for therapeutic purposes [11].

In recent years, the CAR-T cells targeting B cell maturation antigen (BCMA) for treating multiple myeloma (MM) have shown dramatic effect in clinical trials [12–14]. Among these targeted therapies, the LCAR-B38M CAR-T is the most noteworthy. Unlike most CAR-T cells designed for targeting one epitope of antigen, the LCAR-B38M targets two epitopes of BCMA using two tandem VHH sequences. However, whether single VHH targeting one epitope has similar potential in CAR-T therapy further needs to be explored.

We immunized an alpaca with the BCMA-Fc fusion protein. Following the protocol (Supplementary Fig. 1A), we got one VHH with a high-binding affinity of 1.1 nM for BCMA, as determined using the Octet RED system (data not shown). After further humanization, we constructed the humanized VHH-human IgG1 Fc fusion plasmid and expressed it in 293T cells. This protein recognized the BCMA overexpressed on K562 cells, and the expression level was comparable to the level detected using a commercial anti-BCMA antibody (55.0% vs. 44.2%, Supplementary Fig. 1B). We performed the membrane protein panel screening and found that the VHH-Fc specifically recognized BCMA (TNFRSF17) (Supplementary Fig. 1C). These results showed that the VHH we screened has a high affinity and specificity for BCMA targeting.

We constructed the *CAR* gene using the humanized VHH sequence linked with CD8α extracellular, transmembrane domain, 4-1BB cytoplasmic domain and CD3ζ cytoplasmic domain (Supplementary Fig. 1D). The T cells were transduced with the lentivirus and cultured for 12 days to prepare the CAR-T. We labeled BCMA CAR-T cells with diluted BCMA-Fc protein and calculated the EC50 as 0.024 μg/mL (0.71 nM, Supplementary Fig. 1E; Supplementary Table 1). Regarding species specificity, the VHH in the CAR molecule recognized the human BCMA protein, as observed using flow cytometry (Supplementary Fig. 1F), similar to the results obtained using ELISA (Supplementary Table 2). We also constructed a CAR molecule fused with GFP at the cytoplasmic domain. Confocal microscopy images showed that the CAR molecule was evenly distributed on the CAR-T cell membrane (Supplementary Fig. 2), unlike the scFv aggregation on the CAR-T cells observed by Long et al. [15].

**Fig. 1:**
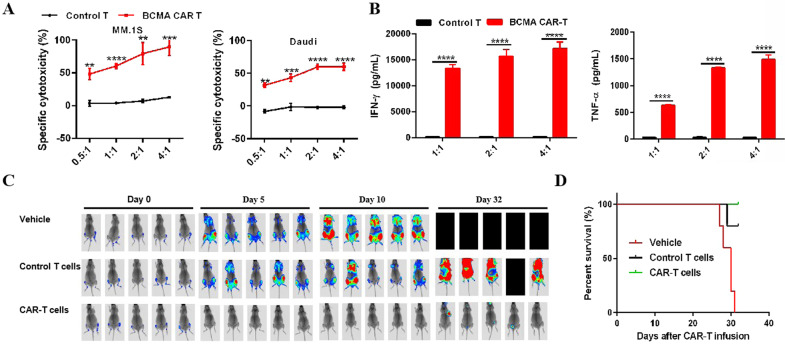
Characterization of anti-BCMA CAR-T cells. **A** BCMA CAR-T could kill the target cells at different ratios of effector:target cells, and killing of MM.1S cells was more efficient than that of Daudi cells, *n* = 3, **P < 0.01, ***P < 0.001, ****P < 0.0001. **B** Secretion of the cytokines IFN-γ and TNF-α during the killing process, *n* = 3, ****P < 0.0001. **C** Bioluminescence image analysis of B-NDG (NOD**-***Prkdc*^*scid*^*IL2rg*^*tm1*^) mice with luciferase gene-transduced MM.1S cells (MM.1S-Luc) that received PBS vehicle control, control T cells, or BCMA CAR-T cells at a dose of 1 × 10^7^ cells/mice in each experimental group at different time points after treatment. **D** CAR-T cells efficiently attenuated the tumor progression and significantly prolonged survival in mice.

To evaluate the ability of BCMA CAR-T to kill tumor cells, we selected MM.1S (BCMA high level) and Daudi (BCMA low level) cells as target cells (Supplementary Fig. 3). The results showed that BCMA CAR-T could kill MM.1S cells more potently than the Daudi cells (Fig. 1A). During the killing process, IFN-γ and TNF-α, levels were increased dramatically in BCMA CAR-T cells compared to those in the control T cells (Fig. 1B). We also performed a proliferation assay, supplementing fresh MM.1S cells into the mixture of BCMA CAR-T cells and MM.1S tumor cells (different ratios) every day for 3 days, and found that tumor cells stimulated BCMA CAR-T cell proliferation, and an increase in the number of tumor cells led to the increased proliferation of CAR-positive cells (Supplementary Fig. 4).

We also carried out in vivo murine experiments. MM.1S cells were transduced with the luciferase gene (MM.1S-Luc) and were inoculated into B-NDG (NOD-*Prkdc*^*scid*^*IL2rg*^*tm1*^) mice. After 14 days of tumor growth, the BCMA CAR-T cells were administered at a dose of 1 × 10^7^ cells/mice; simultaneously, the PBS vehicle control and control T cells were administered to the other two groups (*n* = 5). Using bioluminescence imaging, we observed that BCMA CAR-T cells effectively eliminated the tumor cells in mice. By day 32 after CAR-T administration, all mice in the vehicle group died, whereas all the mice in the CAR-T treated group survived (Fig. 1C, D).

Between April 10, 2018, and June 14, 2019, a total of 36 consecutive patients were enrolled for our study and underwent leukapheresis (Supplementary Fig. 5). Two of these patients underwent leukapheresis but discontinued their participation in the study owing to disease progression before CAR-T infusion. Thus, the results presented are based on the data from 34 patients who received BCMA CAR-T cells at doses of 2.5 × 10^6^ and 10 × 10^6^ CAR-T cells/kg (Supplementary Table 3). The manufacturing of CAR-T was successful in 100% of the patients. The CAR-T cells were composed of a variable proportion of CD4 and CD8 T cells, with a median of 37% (range, 9.5–69.5) CD4^+^ T cells and 61.4% (range, 29.5–90.7) CD8^+^ T cells (Supplementary Fig. 6). Of the 34 patients, one discontinued follow-up after the day 28 efficacy assessment of partial response (PR). The remaining 33 patients were followed up until relapse after stringent complete response (sCR), progressive disease, or death, according to the established procedure.

**Fig. 2:**
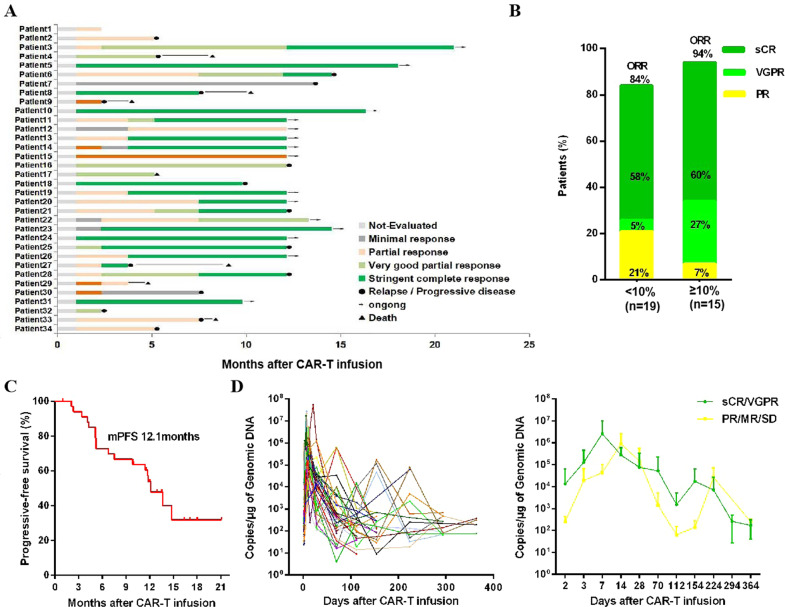
Clinical response in patients. **A** The swimmer’s plot showing clinical response of stringent complete response (sCR), very good partial response (VGPR), partial response (PR), minimal response (MR), stable disease (SD), relapse/progressive disease (PD), or death after BCMA CAR-T cell infusion, according to the International Myeloma Working Group consensus (2016). **B** Objective response rate (ORR, including PR, VGPR, and sCR) was analyzed based on two subgroups of plasma cells (<10% or ≥10%) in the bone marrow. **C** The curve shows progression-free survival (PFS) rates of 53.7% at 12 months and 31.8% at 18 months, respectively. Median PFS was 12.1 months. **D** CAR vector copies in each patient’s peripheral blood were assessed with quantitative PCR at different time points post infusion (left panel). The average copies at different time points were retro analyzed based on the two subgroups of clinical efficacy, sCR/VGPR, or PR/MR/SD (right panel). The sCR/VGPR subgroup shows earlier increased peak of copies.

Based on the International Myeloma Working Group criteria (2016), in 34 patients, we observed a best overall response rate (ORR) of 88.2% (30/34), sCR rate of 55.9% (19/34), very good partial response (VGPR) rate of 17.6% (6/34), and PR rate of 14.7% (5/34) (Fig. 2A). Based on the tumor burden of plasma cells in the bone marrow, the patients were analyzed in two subgroups. The subgroup with plasma cells <10% showed an ORR of 84%, an sCR rate of 58%, a VGPR rate of 5%, and a PR rate of 21%, while the subgroup with plasma cells ≥10% displayed an ORR of 94%, an sCR rate of 60%, a VGPR rate of 27%, and a PR rate of 7% (Fig. 2B). These findings suggested that the patients with bone marrow plasma cells ≥10% may show better response. However, response appeared to be independent of tumor BCMA expression (Supplementary Fig. 7).

The median follow-up time was 12.5 months. The overall survival was 78.8% at 12 months and 18 months (95% confidence interval [CI], 60.5–89.3) (Supplementary Fig. 8). The progression-free survival (PFS) was 53.7% at 12 months (95% CI, 35.3–69.1) and 31.8% at 18 months (95% CI, 12.3–53.5), and the median PFS was 12.1 months (95% CI, 10.1–14.2) (Fig. 2C). Out of seven patients with extramedullary disease, four (57.1%) showed sCR and three showed VGPR, PR, or stable disease (SD), respectively (Supplementary Table 3). Pleural effusion in patient 8 disappeared after 2 months (Supplementary Fig. 9).

Upon detecting CAR vector copies, we found that BCMA CAR-T persisted for a longer time (above the LLOQ, 50 copies/μg genomic DNA). We also found that the copy number increased to peak level earlier in the sCR/VGPR response group (days 7 and 154) compared to that in the PR/minimal response/SD group at days 14 and 224, separately (Fig. 2D).

There was no specific toxicity observed in response to BCMA CAR-T administration (Supplementary Table 4). Cytokine release syndrome (CRS) was the most common adverse event in the treatment. A total of 29 patients (85.3%) had CRS, which was of grade 1 or 2 in 28 patients (82.3%) and grade 3 in 1 patient (2.9%); there were no cases of CRS of grade 4 or higher (Supplementary Table 5). Most patients had elevated levels of cytokine secretion, serum C-reactive protein, and serum ferritin during CRS (Supplementary Fig. 10). CRS occurred early after the CAR-T cell infusion, with a median time of onset of 1 day (range, 1–13) and a median duration of 4 days (range, 2–17). There was no neurotoxicity observed. We found that patients with a baseline plasma cell percentage over 5% tended to have higher levels of IL-6 and IFN-γ (Supplementary Fig. 11); these patients needed to be observed intensively to avoid severe adverse events. Patients who had objective responses (PR or better) did not necessarily have CRS and there was no relation between CRS and BCMA expression (Supplementary Fig. 12).

We, for the first time, report that CAR-T using only one VHH is safe and effective for the clinical treatment of malignancy. Our single VHH-directed BCMA CAR-T cells were safe for the treatment of MM patients and caused sustained remissions.

The bb2121 CAR-T, initially developed by Bluebird Bio, showed an ORR of 85% and sCR/CR rate of 45% in the treatment of 33 patients [12]. LCAR-B38M, developed by Legend Biotech, had an ORR of 88% and a CR rate of 68% in one study that treated 57 patients [14]. JNJ-4528 (same as LCAR-B38M), co-developed by J&J and Legend Biotech in the United States, showed an ORR of 100% and sCR rate of 86%. Whether our single VHH BCMA CAR-T is as good as bb2121 or LCAR-B38M and has the potential to induce long-term durable remissions, will require longer follow-up with the registered patients for ongoing trials.

We also acknowledge some limitations of our study. For example, the patients enrolled in our study were not treated with daratumumab, which was approved in China in July 2019, while the bb2121 and JNJ-4528 trials had patients treated with daratumumab. This may cause incomparability of efficacy and safety among the trials. Another limitation is that further studies need to be conducted to evaluate whether the use of only one humanized VHH in CAR-T, as in our study, can reduced immunogenicity.

In conclusion, our findings clearly demonstrated the promising efficacy and safety of single VHH-directed BCMA CAR-T cells, supporting further development of this BCMA CAR-T for clinical trial and application.

## Supplementary information

Final Supplementary Materials
