# Supplementary material for: Single VHH-directed BCMA CAR-T cells cause remission of relapsed/refractory multiple myeloma
Source: Leukemia. 2021 May 24;35(10):3002–6. doi: 10.1038/s41375-021-01269-3 (PMC8478646; doi:10.1038/s41375-021-01269-3)
Supplement: Supplementary file 1 — Final Supplementary Materials [file 41375_2021_1269_MOESM1_ESM.doc]

**SI Appendix**

**Single VHH-directed BCMA CAR-T cells cause remission of relapsed/refractory multiple myeloma**

Lu Han, Ji-Shuai Zhang, Jian Zhou, Ke-Shu Zhou, Ben-Ling Xu, Lin-Lin Li, Bai-Jun Fang, Qing-Song Yin, Xing-Hu Zhu, Hu Zhou, Xu-Dong Wei, Hong-Chang Su, Bing-Xiang Zhang, Ya-Nan Wang, Bin Xiang, Quan-Li Gao, Yong-Ping Song

**Methods**

***Preparation of K562-BCMA***

K562 (ATCC) cells were cultured in RPMI 1640 with 10% FBS. K562-BCMA cells were produced from K562 cells by lentiviral transduction of the BCMA gene under the control of the CMV promoter along with puromycin selection marker. After single-cell sorting by flow cytometry, the highest BCMA expression clone was selected and maintained in RPMI 1640 media supplemented with 10% FBS.

***Immunization, phage display, and antibody screening***

An adult, healthy alpaca was immunized with BCMA-Fc protein mixed with Freund's adjuvant (Sigma-Aldrich, St. Louis, MO, USA). After six rounds of immunization every two weeks, we measured the concentration of anti-BCMA antibodies in serum with ELISA experiments. When the solution presented a titer higher than pre-immunization levels, we collected lymphocytes for RNA extraction. We constructed a phage display library using the RNA as template for real-time PCR and amplifying the VHH gene sequence. Afterwards, we enriched the anti-BCMA VHH displayed on the phage with three rounds of panning. The gene sequences were subsequently transferred into the prokaryotic expression vector. We used ELISA and flow cytometry to screen candidate sequences, and Octet RED system (ForteBio, CA, USA) to measure the affinity. We selected one sequence as the VHH for recognizing the BCMA in the CAR molecule construction.

***Construction of anti-BCMA CAR and lentivirus production***

We humanized the selected high affinity of VHH by replacing several amino acids with homologous human heavy chain in the framework region. We linked a fragment containing the signal peptide of CD8α with humanized VHH gene and another fragment. The later fragment consisted of CD8α extracellular and transmembrane domain, 4-1BB cytoplasmic domain and CD3ζ cytoplasmic domain, and was synthesized by Igebio (Guangzhou, China). To combine these fragments into one CAR gene, we used a nest-PCR. The CAR gene was cloned into the lentivirus transfer plasmid, a self-inactivation plasmid (Pre-SIN, Pregene Biopharma, Shenzhen, China). The lentivirus carrying the anti-BCMA CAR gene was encased using transfer plasmid, packaging plasmid (psPAX2), and envelope plasmid (pMD2.G) through transiently transfecting suspended 293TS cells (habituated from HEK293T cells, ATCC). After concentration of the crude lentivirus vector, we purified it using hollow fiber (Spectrum, USA) and Core 700 (GE, USA). The purified vector was stored in 0.9% NaCL with human albumin as a stabilizer at -80℃.

***Production of CAR-T cells***

We separated peripheral blood mononuclear cells from the blood drawn from the volunteers’ cubital vein with Ficoll (Sigma-Aldrich, St. Louis, MO, USA). T cells were purified with CliniMACS CD3 reagent (Miltenyi, Bergisch Gladbach, Germany). We used pre-coated RetroNectin (20 μg/mL, GMP grade, Takara Bio Inc, Japan) and anti-CD3 antibody (5 μg/mL, GMP grade, Takara Bio Inc, Japan) on a 6-well plate (Corning Inc, USA) in X-VIVO 15 medium (Lonza, Basel, Switzerland) supplemented with IL-2 (1000 IU/mL, Shandong Quangang Pharmaceutical Co. Ltd., Shandong, China) and 3% autologous serum to activate the T cells. On the next day, they were transduced by adding the lentivirus vectors into the medium at a 3 MOI. Based on the cell density adjustment, we expanded the CAR-T cells by supplementing the X-VIVO 15 medium with IL-2 (1000 IU/mL) and 1% autologous serum. On day 12, we harvested the CAR-T after centrifugation, in the suspended stabilizer solution (of 0.9% NaCL and 0.2% human albumin). We infused fresh CAR-T cell suspension into patients within 8 hours of production. The release criteria included the cell number (adjusted body weight), viability (＞80%), CAR positive percentage (＞10%), CD3 positive percentage (＞95%), vector copies/cell (＜5 copies/cell), endotoxin level (＜10 EU/mL), sterility (in-process control, negative), and mycoplasma (real-time PCR, negative).

***T-cell proliferation assay***

CAR-T cells proliferation in response to BCMA-expressing target cells was performed by co-culturing CAR-T cells with MM.1S target cells at different E:T ratios for 3 days. Briefly, CAR-T cells were washed and suspended at 1×10^6^ cells/mL in T cell media without IL-2. One million CAR-T cells were combined with target cell MM.1S in the 6 well plate. We supplemented same number of fresh MM.1S cells every day. After incubation for three days, co-cultured cells were counted, cells were then labelled with anti-CD3 antibody (FITC，BD) and measured by flow cytometry to determine CD3^+^ cell positivity. CAR positivity were detected using the human BCMA-Fc fusion protein.

***Flow cytometry***

CAR, CD3, BCMA positivity on T cells or tumor cells were detected by flow cytometry. We collected 1 × 10^6^ cells/reaction and divided them into two tubes: one as negative control and the other as treatment with added antibody or BCMA-Fc fusion protein binding the corresponding molecule. For CAR positivity, the BCMA-Fc fusion protein (ACRO, Beijing, China) was used, which was recognized by CAR molecule on the CAR-T cells. For CD3, CD4, CD8, or BCMA expression, anti-CD3 antibody, anti-BCMA antibody, anti-CD4 antibody and anti-CD8 antibody (BioLegend, San Diego, CA, USA) were used. FACS analysis was performed using a FACSCanto II system and CellQuest software (BD Biosciences).

***Cytotoxicity assay***

In order to evaluate the ability of the CAR-T cells to kill the target BCMA-expressing tumor cells, we performed a cytotoxicity assay: MM.1S (multiple myeloma cell line, ATCC), Daudi (lymphoma cell line, ATCC), or K562-BCMA (erythroleukemia cell line, ATCC, with a foreign overexpressing of BCMA). The CAR-T effector and target cells were incubated at different ratios. After 18 h, we collected the supernatants and detected the lactase dehydrogenase released from dead cells using a kit (Promega, Madison, WI, USA).

***Enzyme-linked immunosorbent assay***

CAR-T cells or untransduced T cells were combined with MM.1S tumor cells at different E:T ratios in triplicate wells of a 96-well plate. Following the incubation, cytokines of IFN-γ and TNF-α were measured in supernatants collected from 19 hour co-cultures, using enzyme-linked immunosorbent assay (Dakewe Biotech, Shenzhen, China), according to the manufacturer’s instructions. Plates were read at a wavelength of 450 nm and the results for each cytokine were calculated in pg/mL based on the concentration of standards. Samely, the cytokine level from the serum in clinic, were measured with the same kits.

***Xenograft experiment***

Animal experiments were approved by the Animal Care and Use Committee of JOINN Laboratories (Suzhou, China). Female B-NDG mice (NOD-*Prkdc^scid^IL2rg^tm1^*, Biocytogen, Beijing, China) were inoculated through the tail vein with MM.1S-luc (expressing luciferase gene) cells (1.5 × 10^6^/mouse). After 14 days, the mice were administered PBS (vehicle), control T cells (same number of T cells with CAR-T), or BCMA CAR-T cells (1.0 × 10^7^/mouse) through the tail vein (day 0). The mice were imaged using an in vivo bioluminescence imaging system (IVIS, PerkinElmer, USA) at day 0 (before CAR-T administration), 5, 10, and 32 (after CAR-T administration). On day 32, we euthanized mice in the vehicle group and mice in the other groups after imaging.

***Trial design***

The trial, an open-labeled and investigator-initiated clinical trial (Ethics approval by the institutional review boards of the Henan Cancer Hospital; NCT03661554 (clinicalTrials.gov)), aimed to report the safety of BCMA CAR-T, and its preliminary efficacy against relapsed/refractory (R/R) multiple myeloma (MM). All enrolled patients gave informed consent. We checked previous BCMA expression level on patients’ plasma cells, but it was not strictly required as an enrolling criterion, given the difficulty in determining BCMA expression in patients with low bone marrow plasma cell levels. Eligibility criteria included: (1) measurable tumor burden, defined by the presence of monoclonal protein (M protein) in serum, or serum-free light chain (FLC), or increased bone marrow plasma cells, or extramedullary disease; (2) the patient needed to have received three previous lines of therapy, including immunomodulatory drug or proteasome inhibitor, each line of treatment with at least one complete treatment cycle; (3) the patient had adequate organ function.

To prepare the BCMA CAR-T cells, we drew patients’ peripheral blood according to Good Manufacturing Practices in the immunology laboratory of the Affiliated Cancer Hospital of Zhengzhou University. Lymphodeletion was performed with cyclophosphamide (300–600 mg/m^2^ on days -5, -4) and fludarabine (25–30 mg/m^2^ on days -5, -4, -3). On day 0, fresh CAR-T cells were infused intravenously into patients. On days -2, -1, we checked baseline characteristics. Clinical and laboratory observations were performed within 28 days after infusion to monitor and evaluate its safety. The follow-up time was 2 years (Supplemental Figure 5). Patients did not receive any maintenance therapy post CAR-T cells infusion. The data cut-off for analysis was April 30, 2020.

***Evaluation of safety and clinical response***

On day 0, we collected data on adverse events caused by BCMA CAR-T infusion. Toxicity grade was determined according to Common Terminology Criteria for Adverse Events version 4.03. The cytokine release syndrome (CRS) was graded according to the criteria published by Lee et al^[1]^. The clinical response was evaluated according to the International Myeloma Working Group (2016) consensus, based on data of bone marrow plasma cells, M protein, serum FLC, immunofixation electrophoresis of serum and urine, or computed tomography scan for extramedullary disease.

***Detection of vector copies with quantitative PCR***

Vector copies were detected by analyzing the copies/cell (CAR-T release criteria) or monitoring the CAR gene vector copies in peripheral blood through checking the WPRE element using quantitative PCR. To prepare the standard curve, we used the transfer plasmid including the BCMA CAR gene mixed with 293T genomic DNA. Genomic DNA templates (200 ng) from samples were used for quantification in duplicate. DNA was extracted using a QIAamp DNA Blood Mini Kit (Qiagen, Hilden, Germany). We used one pair of primers and Taq-man in real-time PCR amplification (Fast 7500, Applied Biosystems, USA).

***Statistical analysis***

Data were analyzed with software GraphPad Prism 6.0 and presented as mean ± standard deviation. Missing data were not imputed unless otherwise specified. Survival statistics were analyzed using the Kaplan-Meier method. Association between percentage of plasma cells and interleukins was evaluated using the Mann-Whitney test. *P* value < 0.05 was considered statistically significant.

**Results**

**Supplemental Figure 1. Characterization of anti-BCMA VHH antibody. A** Flow chart of VHH production. **B** The anti-BCMA VHH-Fc fusion protein recognized the BCMA overexpressed on the K562 cells. The expression level was comparable to the level detected by anti-BCMA commercial antibody (55.0% vs 44.2%). **C** VHH-Fc specifically recognizes BCMA (TNFRSF17) by membrane protein panel screening. **D** Diagram of BCMA CAR design, composed of BCMA binding domain (humanized VHH), human CD8α hinge and transmembrane domain, human 4-1BB cytoplasmic domain, and human CD3ζ cytoplasmic domain. **E** The binding affinity of VHH in the CAR molecule on the CAR-T cell membrane. BCMA-Fc was used to measure binding ability using flow cytometry. **F** The VHH in CAR molecule recognizes the human BCMA protein with high-binding ability and the rat BCMA protein with low-binding ability, as determined with flow cytometry.

**
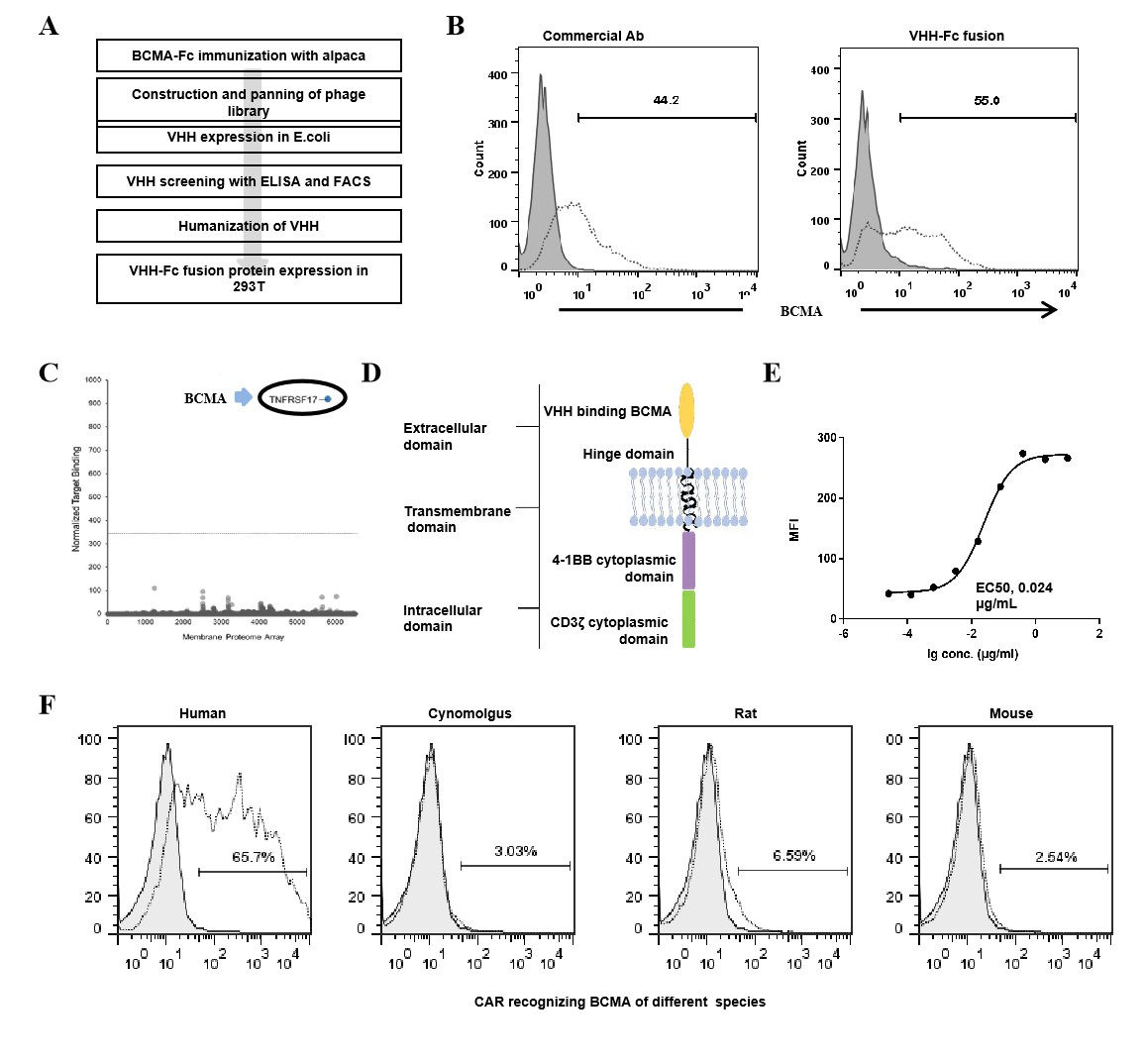
**

**Supplemental Figure 2.** **The CAR molecules were evenly distributed on the BCMA CAR-T cell membrane.** The CAR molecule was linked with GFP reporter protein through G4S linker at the cytoplastic portion. The cofocal microscopy images were taken after CAR-T preparation using the CAR-GFP fusion lentivirus.

**
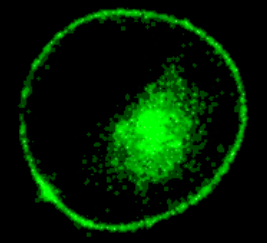
**

**Supplemental Figure 3. Flow cytometry analysis revealed that MM.1S cells expressed high levels of BCMA and Daudi cells expressed low levels of BCMA.**

**
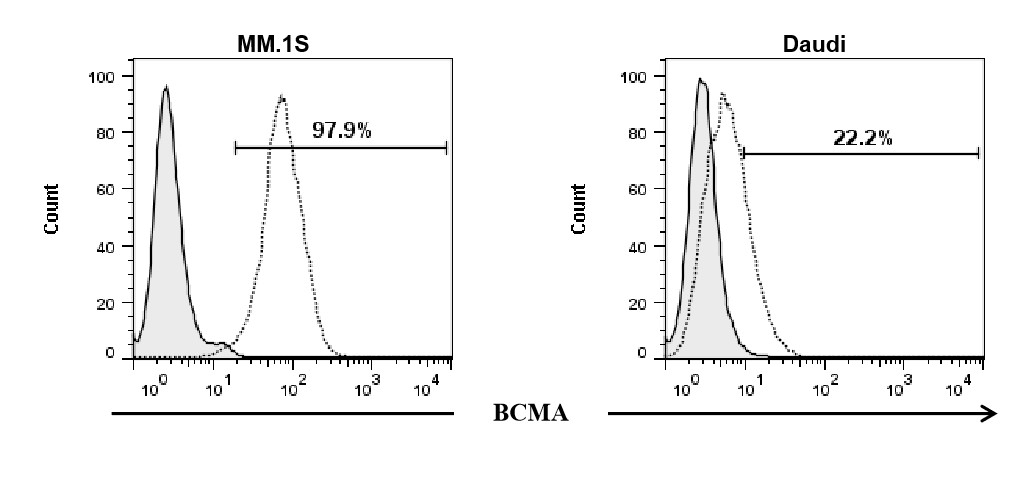
**

**Supplemental Figure 4. The proliferation of CAR**-**T cells stimulated by tumor cells.** Three batches of CAR-T (C1801-1903059, C1801-1903060, C1801-1903061) were incubated with MM.1S cells. The CAR negative cells proliferated with the same speed between the groups with or without tumor cell stimulation, the CAR positive cells proliferated dramatically after tumor cells stimulation.


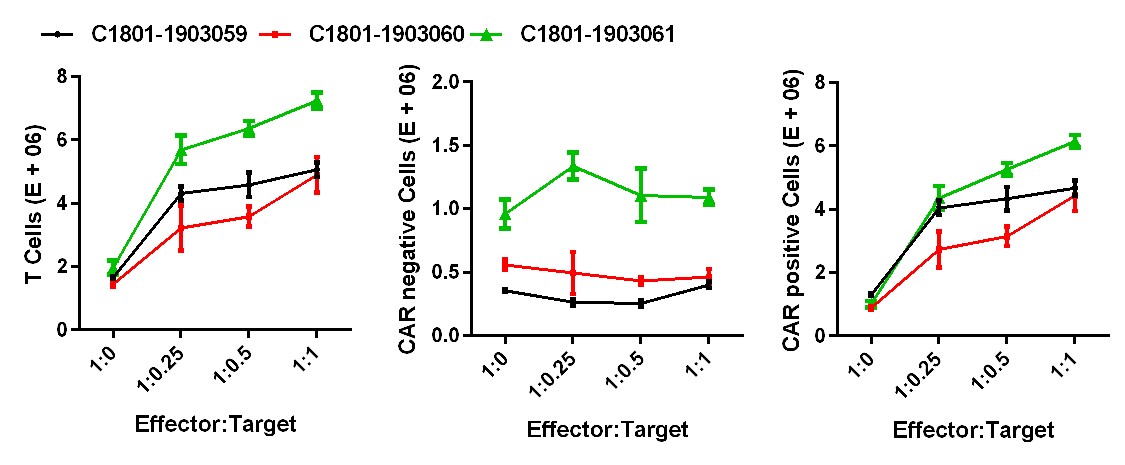


**Supplemental Figure 5. Clinical treatment protocol.** Patients underwent lymphocyte isolation to obtain peripheral blood lymphocytes on day –12, and cells were transduced, cultured, and expanded. The first day of CAR-T infusion was established as day 0, and patients underwent fludarabine/cyclophosphamide-based lymphodepletion chemotherapy on day –5. On weeks 4, 10, 16, 22, and every 10 weeks thereafter, efficacy was evaluated. The patients were followed up for 2 years.

**
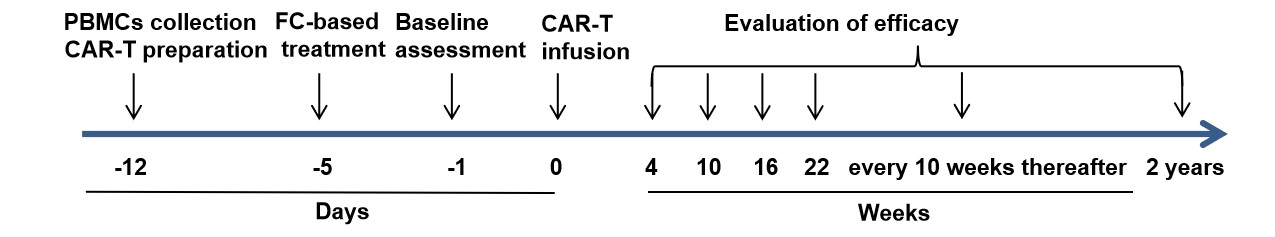
**


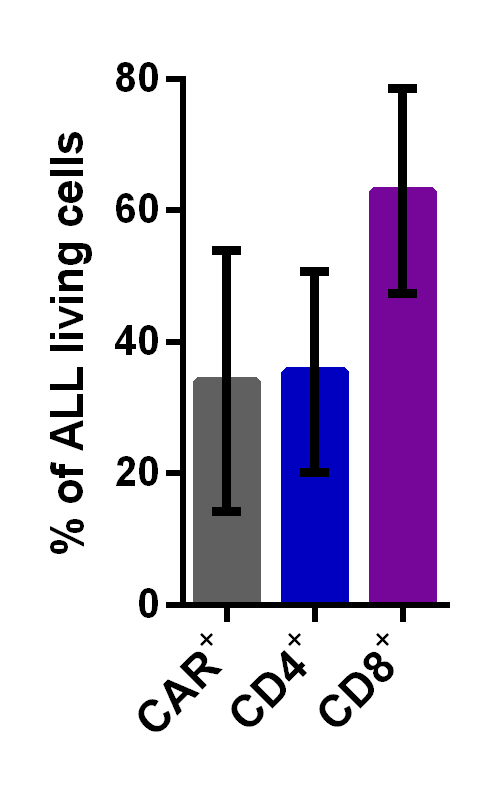
**Supplemental Figure 6. Information of CAR-T cells in product.** The CAR-T cells were composed of a variable proportion of CD4^+^ and CD8^+^ T cells, with a median of 37% (range, 9.5% to 69.5%) CD4^+^ T cells and 61.4% (range, 29.5% to 90.7%) CD8^+^ T cells. The CAR positive percentage of BCMA CAR-T cells was (34.04 ± 19.55)%.

**Supplemental Figure 7. The relationship between response to BCMA expression of plasma cells.**

Response appeared to be independent of tumor BCMA expression. sCR: Stringent complete response; CR: Complete response; VGPR: Very good partial response; PR: Partial response; MR: Minimal response; SD: Stable disease; NS: No statistical difference.

**
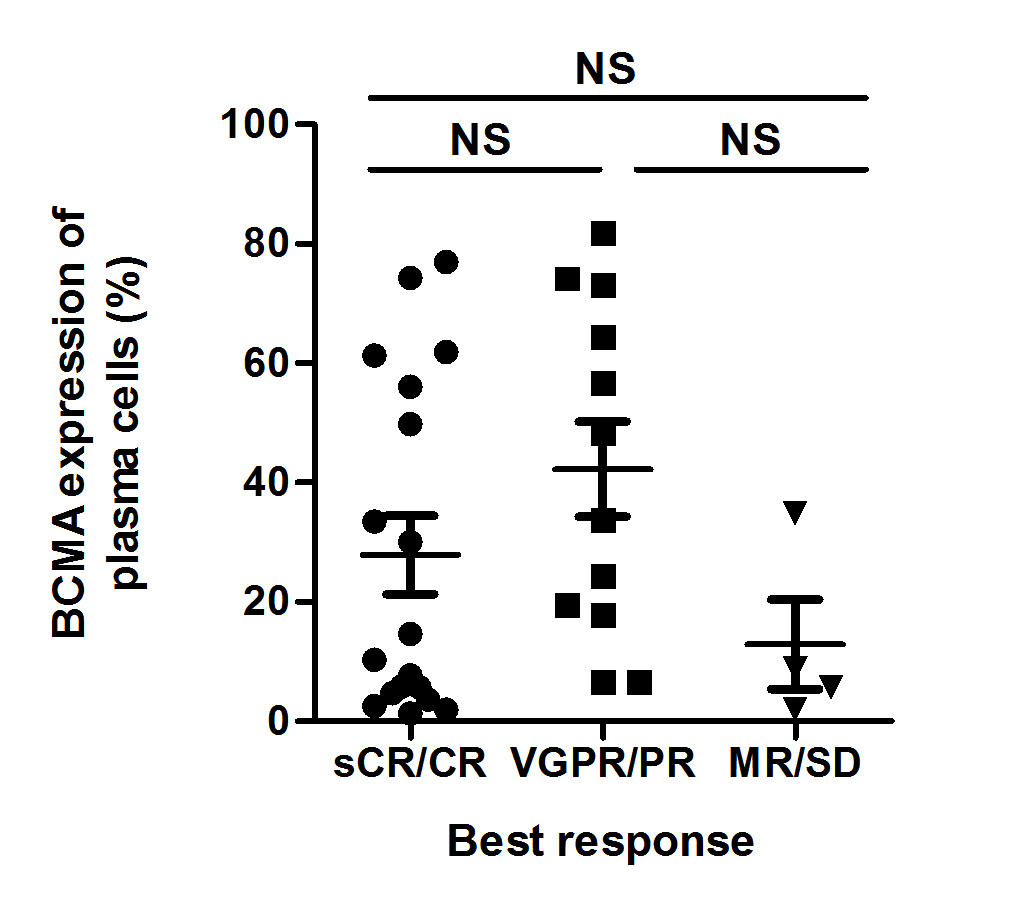
**

**Supplemental Figure 8. The curve showed overall survival data censored at the time of the last follow-up.** The overall survival were 78.8% at 12 months and 18 months**.**

**
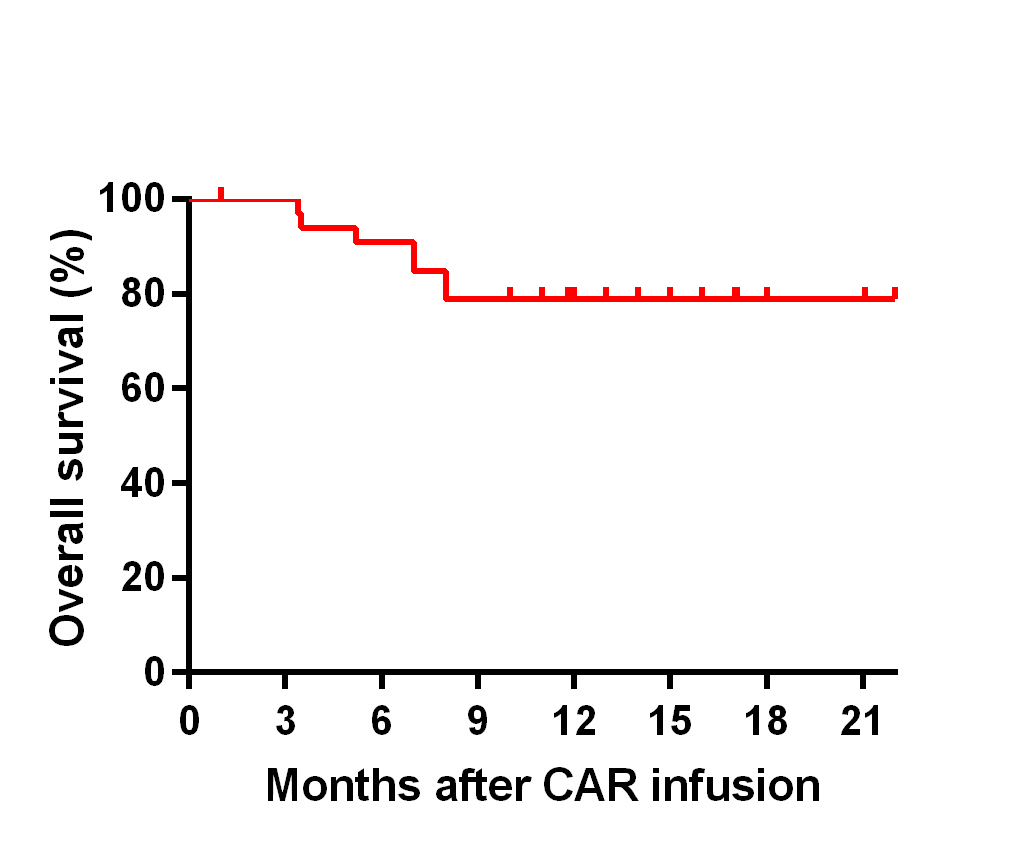
**

**Supplemental Figure 9. Response of extramedullary infiltration lesions in a representative case (patient 8) after CAR-T cell infusion**. The pleural effusion in the chest was slightly increased at 7 days and then disappeared at 2 months and 7 months, as demonstrated by computed tomography. Red arrows indicate sites of tumor lesions.


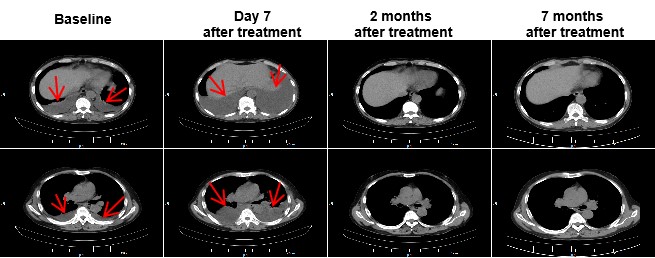


**Supplemental Figure 10. Fold changes (peak concentration compared with baseline) in the levels of serum cytokines (IL-6, IL-10, and IFN-γ), C-reactive protein (CRP), and ferritin after CAR-T cell infusion.**





**Supplemental Figure 11. Cytokine secretion levels according to the baseline plasma cell percentage in the bone marrow (＜5% or ≥5%)**. IL-6 and IFN-γ levels increased in patients with a plasma cell percentage ≥ 5%, statistically, *P* < 0.05.

**
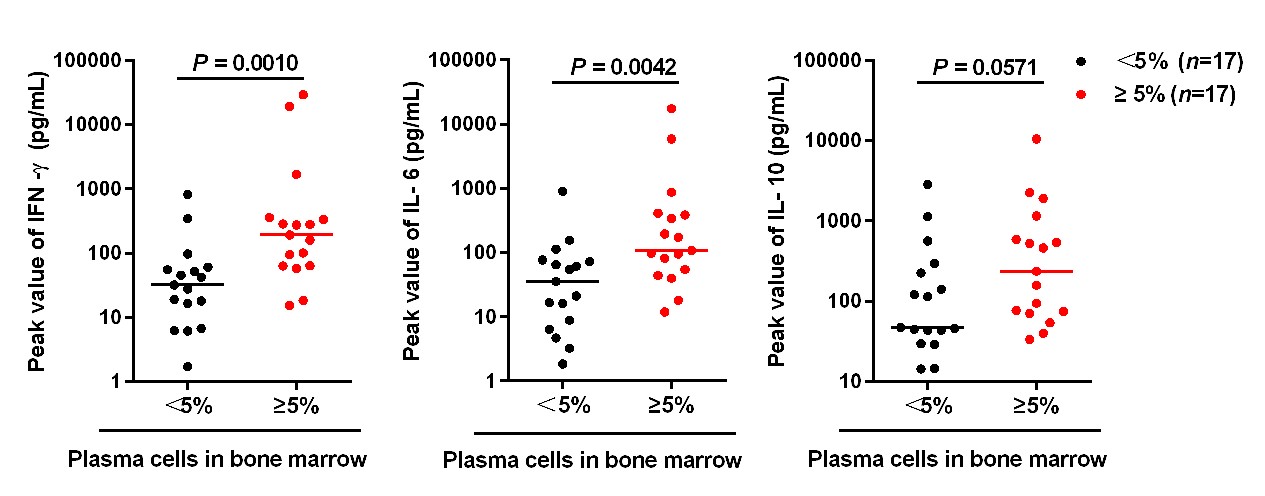
**

**Supplemental Figure 12. The relationship between clinical response and CRS. A** The patients who had a response (partial response or better) did't have CRS necessarily. **B** there was no relation between CRS and BCMA expression. CRS: cytokine-release syndrome; BCMA: B cell maturation antigen; NS: No statistical difference; * *P* <0.05; **** *P* <0.0001.


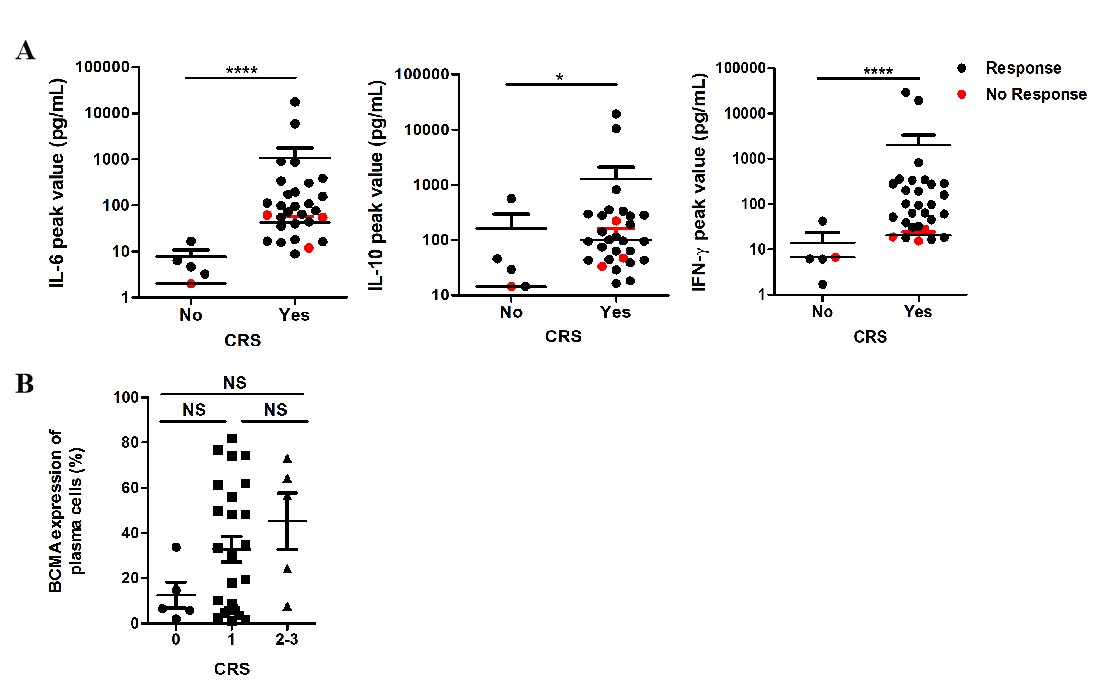


Supplementary Table 1. MFI value measured by FACS through CAR-T binding with human BCMA-Fc.

| Conc. | MFI |
| --- | --- |
| 10 µg/mL | 266 |
| 2 µg/mL | 264 |
| 0.4 µg/mL | 274 |
| 0.08 μg/mL | 219 |
| 0.016 μg/mL | 128 |
| 0.0032 μg/mL | 78.8 |
| 0.00064 μg/mL | 52.2 |
| 0.000128 μg/mL | 40 |
| 0.0000256 μg/mL | 41.8 |

MFI: Fluorescence intensity; FACS: Fluorescence activated cell sorter; CAR: Chimeric antigen receptor;

BCMA: B cell maturation antigen.

Supplementary Table 2. VHH-Fc recognition specificity for BCMA of different species. The BCMA

were coated, then were detected by serial diluted VHH-Fc with ELISA.

| Conc. | OD450 nm | | | |
| --- | --- | --- | --- | --- |
|  | Human | Cynomolgus | Rat | Mouse |
| 2 μg/mL | 3.464 | 0.201 | 0.556 | 0.211 |
| 0.4 μg/mL | 3.638 | 0.102 | 0.259 | 0.122 |
| 0.08 μg/mL | 3.499 | 0.093 | 0.124 | 0.097 |
| 0.016 μg/mL | 2.874 | 0.093 | 0.100 | 0.109 |
| 0.0032 μg/mL | 1.356 | 0.099 | 0.119 | 0.111 |
| 0.00064 μg/mL | 0.469 | 0.094 | 0.108 | 0.108 |
| 0.000128 μg/mL | 0.169 | 0.107 | 0.096 | 0.111 |
| 0.0000256 μg/mL | 0.116 | 0.102 | 0.090 | 0.119 |

VHH: Variable domain of heavy chain of heavy-chain; BCMA: B cell maturation antigen.

Supplementary Table 3. Clinical data, dosage of CAR-T cells, and response in 34 relapsed/refractory multiple myeloma patients.

| Patient | Age/Sex | Subtype | DS/ISS staging | No. of prior lines of therapies | Extra-  medullary disease | PI | IMiD | Tumor burden* | | | | CAR^+^ T cell dosage (cells/kg) | CRS grading | Best response | Follow  up | Use of tocilizumab or glucocorticoid |
| --- | --- | --- | --- | --- | --- | --- | --- | --- | --- | --- | --- | --- | --- | --- | --- | --- |
|  |  |  |  |  |  |  |  | plasma cells in bone marrow (%) | M protein (g/L) | κ/λ | Free light chains (κ, λ) (mg/L) |  |  |  |  |  |
| 1 | 57/M | IgG, κ | IIIA/I | 3 | - | Bortezomib | Lenalidomide | 0.2 | 19.7 | - | - | 1*10^7^ | 0 | PR | -- | - |
| 2 | 57/M | IgG, λ | IIIA/II | 4 | - | Bortezomib | Lenalidomide | 2 | 21.0 | 0.0416 | 6.5, 141.0 | 1*10^7^ | 1 | PR | PD | - |
| 3 | 48/M | IgG, κ | IIIA/II | 5 | - | Bortezomib | No | 38.2 | 51.9 | 13.5892 | 60.2, 4.43 | 1*10^7^ | 2 | sCR | sCR | tocilizumab and glucocorticoid |
| 4 | 53/F | IgA, κ | IIA/II | 5 | - | Bortezomib | Lenalidomide | 5.5 | 18.5 | 0.3065 | 6.56, 21.4 | 1*10^7^ | 1 | VGPR | Death | - |
| 5 | 65/M | IgG, λ | IIIA/I | 7 | - | Bortezomib | Thalidomid | 7 | 7.3 | 0.5861 | 6.74, 11.5 | 5*10^6^ | 1 | sCR | sCR | - |
| 6 | 55/M | IgG, κ | IIIA/I | 7 | Yes | Bortezomib | Thalidomid | 0.4 | 0.0 | 278.0172 | 3225, 11.6 | 5*10^6^ | 1 | sCR | Relapse | - |
| 7 | 43/F | IgG, κ | IIIA/I | 8 | - | No | Thalidomid | 0.2 | 1.8 | 0.7558 | 6.5, 8.6 | 5*10^6^ | 0 | MR | PD | - |
| 8 | 64/M | λ | IIA/I | 3 | Yes | Bortezomib | Lenalidomide | 0 | 0.0 | 0.0745 | 6.5, 87.3 | 5*10^6^ | 1 | sCR | Death | - |
| 9 | 50/M | IgA, λ | IIIA/II | 12 | - | Bortezomib | Thalidomid | 23.2 | 8.0 | 0.0755 | 6.5, 86.1 | 5*10^6^ | 1 | SD | Death | - |
| 10 | 55/M | κ | IIIA/I | 3 | Yes | Bortezomib | Lenalidomide/ Thalidomid | 1.2 | 0.0 | 74.6711 | 1135, 15.2 | 5*10^6^ | 1 | sCR | sCR | - |
| 11 | 63/M | IgG, λ | IIIA/II | 6 | - | Bortezomib | Thalidomid | 0.2 | 3.1 | 0.3675 | 12.2, 33.2 | 2.5*10^6^ | 1 | sCR | sCR | - |
| 12 | 63/M | IgG, κ | IIIA/II | 3 | Yes | Bortezomib | Lenalidomide | 1.4 | 17.8 | 0.6478 | 8.81, 13.6 | 5*10^6^ | 1 | PR | PR | - |
| 13 | 65/F | IgG, λ | IIIA/I | 3 | - | Bortezomib | Thalidomid | 0.5 | 4.8 | 0.7752 | 10, 12.9 | 5*10^6^ | 0 | sCR | sCR | - |
| 14 | 68/M | κ | IIIA/III | 6 | Yes | Bortezomib | Lenalidomide/ Thalidomid | 0.6 | 0.0 | >116.0287 | >4850, 41.8 | 2.5*10^6^ | 1 | SD | SD | - |
| 15 | 56/M | IgG, λ | IIIA/I | 3 | - | Bortezomib | Thalidomid | 0 | 2.9 | 0.4039 | 8.2, 20.3 | 1*10^7^ | 1 | sCR | sCR | - |
| 16 | 51/M | κ | IIIA/II | 3 | Yes | Bortezomib | Thalidomid | 4.0 | 4.6 | >617.0483 | >4850, 7.86 | 5*10^6^ | 1 | VGPR | PD | - |
| 17 | 57/M | IgD, λ | IIIA/III | 3 | - | Bortezomib | Lenalidomide/ Pomalidomide | 85 | 0.0 | 0.4676 | 6.5, 13.9 | 2.5*10^6^ | 1 | VGPR | Death | - |
| 18 | 68/M | κ | IIIA/III | 3 | - | Bortezomib | No | 68.2 | 2.9 | >492.8862 | >4850, 9.84 | 2.5*10^6^ | 1 | sCR | Relapse | - |
| 19 | 59/M | IgG, λ | IIIA/I | 3 | Yes | Bortezomib | No | 0.8 | 6.2 | <0.0016 | 13.4, >3675 | 2.5*10^6^ | 0 | sCR | sCR | - |
| 20 | 73/F | IgG, λ | IIA/II | 10 | - | Bortezomib | Lenalidomide | 69.6 | 39.2 | 0.0264 | 6.5, 246 | 5*10^6^ | 1 | sCR | sCR | - |
| 21 | 51/F | IgG, λ | IIIA/II | 3 | - | Bortezomib | No | 60.8 | 7.3 | 0.686 | 21.6, 315 | 5*10^6^ | 1 | sCR | Relapse | - |
| 22 | 47/M | IgG, κ | IIA/I | 9 | - | Bortezomib | Lenalidomide | 29 | 19.7 | 5.743 | 37.1, 6.46 | 5*10^6^ | 1 | VGPR | VGPR | - |
| 23 | 53/F | IgA, κ | IIIB/III | 4 | - | Bortezomib | Lenalidomide | 0.5 | 17.7 | 198.3696 | 3650, 18.4 | 5*10^6^ | 1 | sCR | sCR | - |
| 24 | 61/F | IgG, λ | IIIA/I | 6 | - | Bortezomib/ Ixazomib | Lenalidomide | 45 | 1.0 | <0.0016 | 6.1, >3755 | 5*10^6^ | 1 | sCR | sCR | - |
| 25 | 59/F | IgA, λ | IIIA/I | 6 | - | Bortezomib | Lenalidomide | 35.2 | 26.1 | 0.0162 | 6.1,377.5 | 5*10^6^ | 1 | sCR | Relapse | - |
| 26 | 69/M | IgA, λ | IIIA/III | 5 | - | Bortezomib | Thalidomid | 32 | 19.8 | 0.0181 | 6.1, 337.5 | 5*10^6^ | 1 | sCR | sCR | - |
| 27 | 47/F | IgA, κ | IIIA/I | 3 | - | Bortezomib | Thalidomid | 3 | 17.2 | 1.3435 | 17.6, 13.1 | 5*10^6^ | 1 | sCR | Death | - |
| 28 | 49/F | IgG, λ | IIIA/III | 4 | - | Bortezomib | Thalidomid | 76.5 | 68.3 | 0.0029 | 6.1, 2105 | 5*10^6^ | 2 | sCR | Relapse | - |
| 29 | 54/F | IgG, κ | IIB/III | 3 | - | Bortezomib | No | 13 | 22.3 | 47.8482 | 422.5, 8.83 | 2.5*10^6^ | 3 | PR | Death | - |
| 30 | 50/F | IgA, λ | IIA/I | 8 | - | Bortezomib/ Ixazomib | Lenalidomide/ Thalidomid | 3.2 | 20.1 | 0.1101 | 12.0, 109 | 5*10^6^ | 1 | MR | PD | - |
| 31 | 63/F | IgG, κ | IIIA/III | 3 | - | Ixazomib | Lenalidomide | 46 | 14.0 | >797.0383 | >4575, 5.74 | 5*10^6^ | 2 | sCR | sCR | tocilizumab and glucocorticoid |
| 32 | 48/M | IgA, κ | IIIA/I | 9 | - | Bortezomib | No | 22 | 0.0 | 1022.7273 | 4500, 4.4 | 5*10^6^ | 1 | VGPR | PD | - |
| 33 | 50/F | IgG, λ | IIA/I | 7 | - | Bortezomib | Lenalidomide/ Thalidomid | 2 | 12.2 | 0.0899 | 21, 223.5 | 5*10^6^ | 0 | PR | Death | - |
| 34 | 63/M | IgG, κ | IIIA/I | 3 | - | Bortezomib | Thalidomid | 40 | 29.1 | 3.2125 | 19.5, 6.07 | 5*10^6^ | 2 | VGPR | PD | - |

M: Male; F: Female; DS: Durie-Salmon staging system; ISS: International staging system; PI: Proteasome inhibitor; IMiD: Immunomodulatory drugs;

CRS: Cytokine-release syndrome; --: discontinued follow-up; -: not applicable.

* Tumor burden: measurable tumor indexes of plasma cell in bone marrow, M protein, and free light chains just before T cell infusion.

Supplementary Table 4. Adverse events of 34 patients (frequency ≥10%)

|  | Adverse events | Frequency (percentage, n=34) | |
| --- | --- | --- | --- |
|  |  | Any grade | ≥ Grade 3 |
|  | Cytokine release syndrome, CRS | 85.3% (29/34) | 2.9% (1/34) |
|  | Increased C-reactive protein | 58.8% (20/34) | 0 |
|  | Thrombocytopenia | 50.0% (17/34) | 38.2% (13/34) |
|  | Neutropenia | 50.0% (17/34) | 44.1% (15/34) |
|  | Leukopenia | 47.1% (16/47) | 32.4% (11/34) |
|  | Iron overload | 41.2% (14/34) | 0 |
|  | Lymphopenia | 38.2% (13/34) | 26.5% (9/34) |
|  | Anemia | 29.4% (10/34) | 20.6% (7/34) |
|  | Hypogammaglobulinemia | 17.6% (6/34) | 0 |
|  | Increased Hydroxybutyrate Dehydrogenase | 14.7% (5/34) | 0 |
|  | Increased lactic dehydrogenase | 14.7% (5/34) | 0 |
|  | Hypokalemia | 11.8% (4/34) | 2.9% (1/34) |
|  | Heart failure | 11.8% (4/34) | 2.9% (1/34) |
|  | Hypoalbuminemia | 11.8% (4/34) | 0 |
|  | Increased creatinine | 11.8% (4/34) | 0 |

Supplementary Table 5. Characteristics and management of cytokine release syndrome.

| Parameter |  | Total  (n = 34) |
| --- | --- | --- |
| Patients with a CRS event-no. (%) |  |  |
| Any grade |  | 29 (85%) |
| Grade ≥3 |  | 1 (3%) |
| Median (min-max) time to onset, days |  |  |
| Any grade |  | 1 (1-13) |
| Grade ≥3 |  | 1 |
| Median (min-max) duration, days |  |  |
| Any grade |  | 4 (2-17) |
| Grade ≥3 |  | 6 |
| Tocilizumab use-no. (%)† |  | 2 (5.9%) |
| Corticosteroid use-no. (%) |  | 2 (5.9%) |

CRS: Cytokine release syndrome.

Uniformly graded per *Lee DW, et al*.

†The decision to give tocilizumab was at the treating physician's discretion based on protocol-specified toxicity

management guidelines.

**References**

1. Lee DW, Gardner R, Porter DL, et al. Current concepts in the diagnosis and management of cytokine release

syndrome. Blood. 2014; 124(2):188-195.
